# Extended Kalman Filter with Reduced Computational Demands for Systems with Non-Linear Measurement Models

**DOI:** 10.3390/s20061584

**Published:** 2020-03-12

**Authors:** Piotr Kaniewski

**Affiliations:** Military University of Technology, ul. gen. S. Kaliskiego 2, 00-908 Warszawa, Poland

**Keywords:** Extended Kalman filter, adaptive filter, linearization, nonlinear system model

## Abstract

The paper presents a method of computational complexity reduction in Extended Kalman Filters dedicated for systems with non-linear measurement models. Extended Kalman filters are commonly used in radio-location and radio-navigation for estimating an object’s position and other parameters of motion, based on measurements, which are non-linearly related to the object’s position. This non-linearity forces designers to use non-linear filters, such as the Extended Kalman Filter mentioned, where linearization of the system’s model is performed in every run of the filter’s loop. The linearization, consisting of calculating Jacobian matrices for non-linear functions in the dynamics and/or observation models, significantly increases the number of operations in comparison to the linear Kalman filter. The method proposed in this paper consists of analyzing a variability of Jacobians and performing the model linearization only when expected changes of those Jacobians exceed a preset threshold. With a properly chosen threshold value, the proposed filter modification leads to a significant reduction of its computational burden and does not noticeably increase its estimation errors. The paper describes a practical simulation-based method of determining the threshold. The accuracy of the filter for various threshold values was tested for simplified models of radar systems.

## 1. Introduction

In radio-electronic systems, especially radio-location or radio-navigation systems, a common issue is estimating the position and other parameters of motion of an observed or navigated object [1]. These quantities, treated as elements of a state vector [2], are calculated based on measurements of distances, differences of distances, sums of distances, angles, velocities etc., acquired with the use of various sensors. In most cases, the measured quantities are non-linearly related to the object’s position, and this relationship can be written as a non-linear equation called the measurement model [1,2,3,4,5,6].

Algorithms of object position estimation used in radiolocation and radio-navigation systems are often model-based, which implies the use of a dynamic model to describe the relationship between the future and the current state vector values [2], and enables a future state prediction. The dynamics model equation, similar to the measurement model equation, is non-linear in many radio-electronic systems [4,7].

Due to the common non-linearity of radio-electronic system models, classical linear Kalman filters [1,2] cannot usually be applied for their state estimation and, instead, non-linear filters such as a Linearized Kalman Filter (LKF) [2], an Extended Kalman Filter (EKF) [2,8], an Unscented Kalman Filter - UKF [9], or a Particle Filter (PF) [10,11] are used. Due to its simplicity and versatility, one of the most frequently applied algorithms is the Extended Kalman Filter.

In every computational step of the EKF algorithm, the non-linear equations of the system model are linearized. If both the dynamics and the observation model are non-linear, Jacobians of both non-linear functions must be calculated [2,4]. In many systems, however, only the observation model is non-linear, and then only one Jacobi matrix must be calculated [1,8]. In both considered cases, the additional model linearization performed in every run of the main loop of the algorithm means an increased computational burden in comparison to a linear filter.

To accelerate calculations on platforms with limited computational resources, or to reduce the energy consumption, in engineering practice, various methods of a computational burden reduction were sought. With this aim in mind, the information Kalman filter and sequential Kalman filter were proposed [2]. In the case of the EKFs, in the literature, one can also find solutions consisting of dividing the total system model into linear and non-linear parts, which can be applied in the case of partially non-linear models [12]. In Reference [13], an efficient and robust method of numerical integration of a non-linear continuous dynamics model equation is proposed and it is claimed to be more than two orders of magnitude faster than the method used in standard EKFs. This idea, however, is applicable for continuous-discrete systems and is not useful in fully discrete systems as considered in this paper. A good tutorial on existing code optimization methods that can be used for computational complexity reduction in various Kalman filter extensions is given in Reference [14]. The described methods make use of existing linearities in non-linear system’s models, conditional linearities, special forms of system’s matrices, e.g., their block diagonalities, alternative filter’s formulations, etc. A problem of dealing with real-time constraints in EKF and UKF implementations on low processing power platforms, like microcontrollers, is discussed in Reference [15]. To save the computational cost, the authors suggest rewriting the filter’s equations as a couple of simpler scalar equations and exploiting the sparsity of matrices. Using look-up tables instead of traditional Kalman gains calculations in tracking loops in Global Navigation Satellite Systems (GNSS) receivers is presented in Reference [16], but the utility of this method has been demonstrated in linear filters only. None of the mentioned sources describes a method similar to the one presented in this paper.

The method of computational burden reduction proposed in this paper consists of analyzing a variability of a Jacobi matrix of a non-linear transformation in the measurement model and performing the model linearization only if the expected changes of the elements of this matrix exceed a predefined threshold. As a result, the Jacobian is calculated only in selected steps of the main loop of the EKF algorithm, and the times between its updates are variable and depend on the variability of this matrix estimated by the filter itself. Therefore, the considered algorithm can be classified as an adaptive filter. With appropriately chosen threshold values, the proposed solution significantly reduces computational demands and, at the same time, only slightly reduces estimation accuracy.

The layout of the further parts of this paper is as follows: the classical extended Kalman filter is presented in Section 2, and its modified version aimed at reducing the computational burden is described in Section 3. Section 4 contains a description of a simple radiolocation systems with linear dynamics models and non-linear measurement models, and gives the equations necessary for the implementation of the proposed adaptive solution. The paper also includes selected results of simulations and a practical simulation-based method of determining the threshold in Section 5. Conclusions are provided in Section 6.

## 2. Extended Kalman Filter

This paper considers a simpler case of a system with a linear dynamics model and a non-linear measurement model. However, it is often met in radio-location or radio-navigation practice. Such a model is given by the following equations [2,8,10].

**Figure 1:**
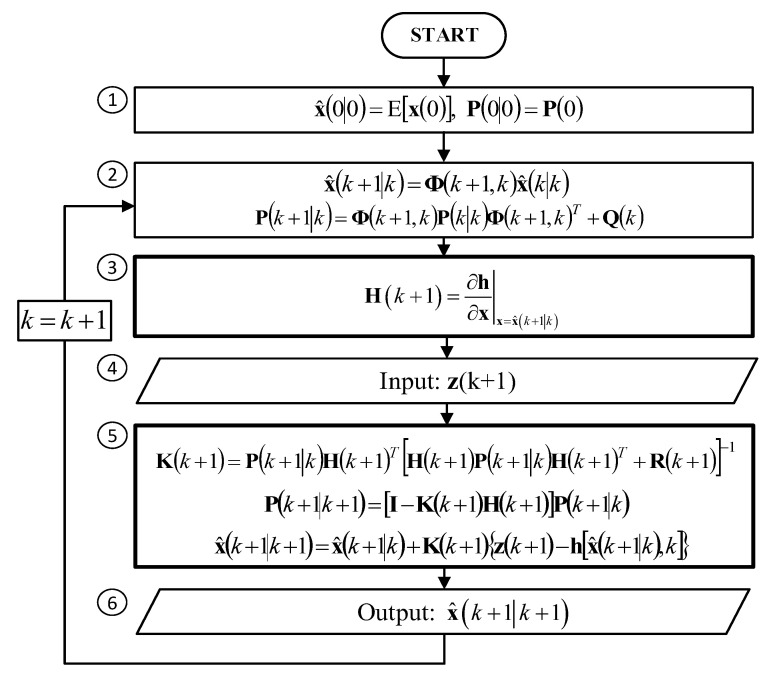
Block diagram of the Extended Kalman Filter.

(1) x(k+1)=Φ(k+1,k)x(k)+w(k),

(2) z(k)=h[x(k)]+ε(k),

where: **x** – state vector, Φ – transition matrix, **w** – vector of process disturbances, **z** – measurement vector, **h**(*) – non-linear measurement function, ε – vector of measurement errors, *k* – index of time t=kT, T – period between two discrete time steps. The EKF algorithm for such a system is shown in Figure 1.

The EKF algorithm contains the following steps:

1. Initialization of the estimated state vector $\hat{x}\left(0|0\right)$ and the covariance matrix of filtration errors P(0|0),
2. Prediction of the state vector $\hat{x}\left(k+1|k\right)$ and calculation of the covariance matrix of prediction errors P(k+1|k),
3. Calculation of the observation matrix H(k+1) which is a Jacobian of the non-linear measurement function **h**(*),
4. Acquisition of a new measurement vector **z**(k+1),
5. Calculation of the Kalman gains matrix K(k+1) and correction of the prediction results, i.e., calculation of the updated state vector $\hat{x}\left(k+1|k+1\right)$ and the covariance matrix of filtration errors P(k+1|k+1),
6. Reporting of the state vector estimate $\hat{x}\left(k+1|k+1\right)$,

An additional step of the EKF in comparison to a linear Kalman filter can be seen in step (3), where a Jacobian matrix of the function **h**(*) is calculated.

The **Q** and **R** matrices in the filter’s equations represent a covariance matrix of process disturbances **w** and a covariance matrix of the measurement noises ε, respectively [2].

## 3. Extended Kalman Filter with Reduced Computational Demands

Assuming that the state vector x is composed of n elements and the measurement vector z contains m simultaneously acquired measurements, Equation (2) can be presented in more detail as follows, where the *k* index was omitted for simplicity.

(3) $$\left[\begin{array}{c} z_{1}\\ z_{2}\\ \begin{array}{c} \vdots \\ z_{m} \end{array} \end{array}\right]=\left[\begin{array}{c} h_{1}\left(x_{1},x_{2},\ldots x_{n}\right)\\ h_{2}\left(x_{1},x_{2},\ldots x_{n}\right)\\ \begin{array}{c} \vdots \\ h_{m}\left(x_{1},x_{2},\ldots x_{n}\right) \end{array} \end{array}\right]+\left[\begin{array}{c} \varepsilon_{1}\\ \varepsilon_{2}\\ \begin{array}{c} \vdots \\ \varepsilon_{m} \end{array} \end{array}\right].$$

Thus, a calculation of the Jacobian for the **h**(*) function requires calculating numerical values of m×n partial derivatives, which compose the H matrix.

(4) $$H=\frac{\partial h}{\partial x}=\left[\begin{array}{ccc} H_{11} & H_{12} & \begin{array}{cc} \cdots & H_{1n} \end{array}\\ H_{21} & H_{22} & \begin{array}{cc} \cdots & H_{2n} \end{array}\\ \begin{array}{c} \vdots \\ H_{m1} \end{array} & \begin{array}{c} \vdots \\ H_{m2} \end{array} & \begin{array}{cc} \begin{array}{c} \ddots \\ \cdots \end{array} & H_{mn} \end{array} \end{array}\right]=\left[\begin{array}{ccc} \frac{\partial h_{1}\left(x_{1},x_{2},\ldots x_{n}\right)}{\partial x_{1}} & \frac{\partial h_{1}\left(x_{1},x_{2},\ldots x_{n}\right)}{\partial x_{2}} & \begin{array}{cc} \cdots & \frac{\partial h_{1}\left(x_{1},x_{2},\ldots x_{n}\right)}{\partial x_{n}} \end{array}\\ \frac{\partial h_{2}\left(x_{1},x_{2},\ldots x_{n}\right)}{\partial x_{1}} & \frac{\partial h_{2}\left(x_{1},x_{2},\ldots x_{n}\right)}{\partial x_{2}} & \begin{array}{cc} \cdots & \frac{\partial h_{2}\left(x_{1},x_{2},\ldots x_{n}\right)}{\partial x_{n}} \end{array}\\ \begin{array}{c} \vdots \\ \frac{\partial h_{m}\left(x_{1},x_{2},\ldots x_{n}\right)}{\partial x_{1}} \end{array} & \begin{array}{c} \vdots \\ \frac{\partial h_{m}\left(x_{1},x_{2},\ldots x_{n}\right)}{\partial x_{2}} \end{array} & \begin{array}{cc} \begin{array}{c} \ddots \\ \cdots \end{array} & \frac{\partial h_{m}\left(x_{1},x_{2},\ldots x_{n}\right)}{\partial x_{n}} \end{array} \end{array}\right].$$

In practice, the H matrix elements often change only very slightly between successive time steps, and, therefore, their updating in each step *k* results in unnecessary computational burden without significantly improving the filter’s estimation accuracy. In such cases, updating the H matrix elements only in selected steps of the algorithm loop may be considered. The values of derivatives $H_{ij}$, however, display various sensitivities for changes of the state vector elements. Thus, without a priori knowledge of the estimated state vector $\hat{x}$ trajectory, one cannot decide how often the H matrix should be updated. In this paper, an adaptive approach is proposed, consisting of determining the H matrix update times based on the expected changes of the partial derivatives $H_{ij}$ from their last actualization.

A differential $dH_{ij}$, which is a function of the estimated state vector $\hat{x}$, can be written as follows.

(5) $$dH_{ij}=\frac{\partial H_{ij}}{\partial x}d\hat{x}=\left[\begin{array}{ccc} \frac{\partial H_{ij}}{\partial x_{1}} & \frac{\partial H_{ij}}{\partial x_{2}} & \begin{array}{cc} \cdots & \frac{\partial H_{ij}}{\partial x_{n}} \end{array} \end{array}\right]\left[\begin{array}{c} d{\hat{x}}_{1}\\ d{\hat{x}}_{2}\\ \begin{array}{c} \vdots \\ d{\hat{x}}_{n} \end{array} \end{array}\right],$$

In addition, by approximating it with a finite increment, the following is obtained.

(6) $$\Delta H_{ij}\approx \frac{\partial H_{ij}}{\partial x}\Delta \hat{x}=\left[\begin{array}{ccc} \frac{\partial H_{ij}}{\partial x_{1}} & \frac{\partial H_{ij}}{\partial x_{2}} & \begin{array}{cc} \cdots & \frac{\partial H_{ij}}{\partial x_{n}} \end{array} \end{array}\right]\left[\begin{array}{c} \Delta {\hat{x}}_{1}\\ \Delta {\hat{x}}_{2}\\ \begin{array}{c} \vdots \\ \Delta {\hat{x}}_{n} \end{array} \end{array}\right].$$

An expected (linearly extrapolated) relative change of the $H_{ij}$ element from the last update realized at the time step l until the current time step k+1 can be calculated as follows.

(7) $$\frac{\Delta H_{ij}\left(k+1,l\right)}{H_{ij}\left(l\right)}\approx \left[{\frac{\partial H_{ij}}{\partial x}|}_{\hat{x}\left(l|l-1\right)}/H_{ij}\left(l\right)\right]\Delta \hat{x}=M_{ij}\left(l\right)\Delta \hat{x}=M_{ij}\left(l\right)\left[\hat{x}\left(k+1|k\right)-\hat{x}\left(l|l-1\right)\right].$$

In the proposed algorithm, the H matrix is calculated for the first time at the time step k=1. After its first calculation and each subsequent update at the time step l, the $M_{ij}\left(l\right)$ coefficient and the predicted state vector $\hat{x}\left(l|l-1\right)$ are stored. In the steps following the H matrix update, monitoring variables $\left|M_{ij}\left(l\right)\left[\hat{x}\left(k+1|k\right)-\hat{x}\left(l|l-1\right)\right]\right|$ are calculated and compared with a predefined threshold  ρ. If any of such variables exceeds the threshold  ρ, the $H_{ij}$ element of H and a new value of $M_{ij}$ are calculated. The proposed solution eliminates the necessity of calculating the H matrix as a whole and, in every step of the EKF algorithm loop, and therefore it may reduce the number of performed arithmetic operations.

The presented EKF modification reduces the computational load related to the calculation of the H matrix, but it adds additional calculations of $M_{ij}\left(l\right)$ coefficients after every H matrix update and comparisons of monitoring variables $\left|M_{ij}\left(l\right)\left[\hat{x}\left(k+1|k\right)-\hat{x}\left(l|l-1\right)\right]\right|$ with the threshold ρ at each time step k. With a properly chosen threshold, however, this additional computational overhead is smaller than the obtained savings. This is due to the fact that calculating $M_{ij}$ or $H_{ij}$ is of similar complexity, but, in a standard EKF, $H_{ij}$ parameters are calculated at every step k, whereas the coefficients $M_{ij}$ can be calculated much less frequently. The operation of calculating monitoring variables and their comparison with the threshold ρ is admittedly realized at each time step k, but it is a simple operation that does not increase the computational burden significantly.

**Figure 2:**
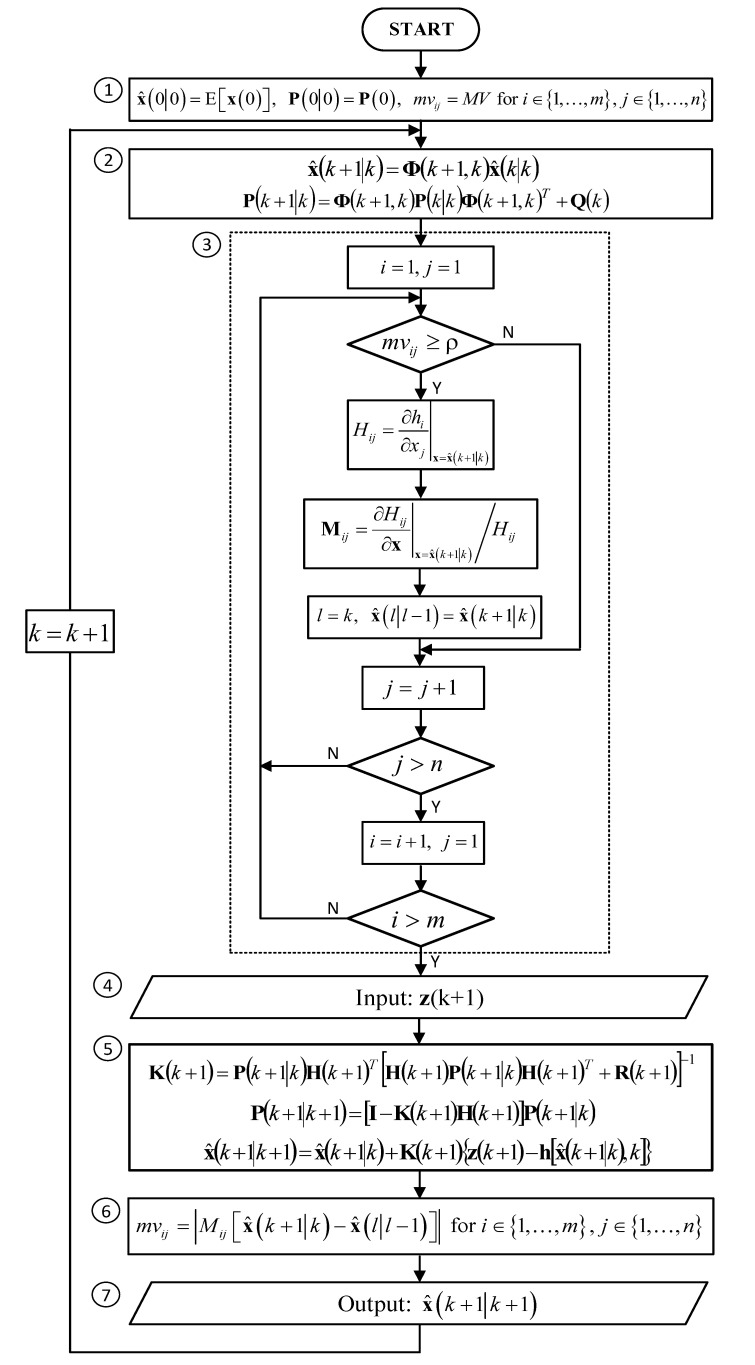
Block diagram of the modified Extended Kalman Filter.

A block diagram of the modified EKF algorithm is shown in Figure 2. The step numbered as 3 in this algorithm replaces the calculation of the total H matrix at every step k of the standard algorithm from Figure 1. The monitoring variables named as $mv_{ij}$ are initially set to large values MV>ρ to ensure that the H matrix is calculated for the first time at the time step k=1. Their values are recalculated at every step k in a block numbered as 6.

## 4. Examples of Application

### 4.1. Range-Only Tracking in 2D Radar

**Figure 3:**
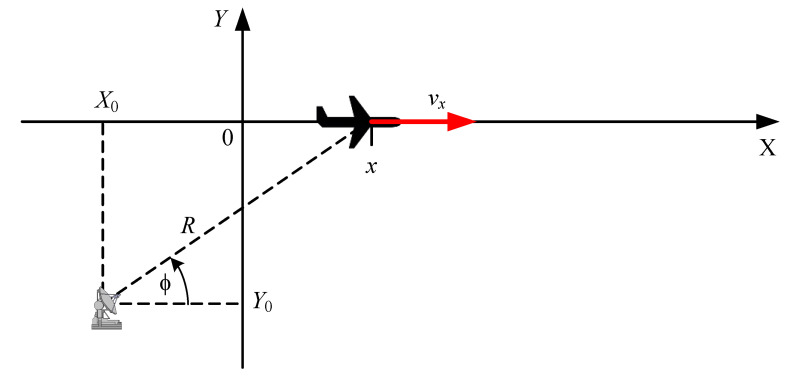
An example of a simple radar system.

The performance of the proposed algorithm was first demonstrated for a simple radar system, graphically presented in Figure 3. The considerations were limited to a 2D case where both the radar and the observed object are in the OXY frame of reference. It is assumed that the object moves in one direction only, along the OX axis, and only ranges R between the radar and the object are measured. The coordinates of the radar are $\left(X_{0},Y_{0}\right)$ and those of the object are (x,y). Such a simple model is enough to present all the important features of the proposed modified EKF algorithm without focusing on the intricacies of models of more realistic radar systems.

A linear uniform motion of the object with disturbances (random acceleration) was assumed. Such a model, called a PV (Position-Velocity) model in the literature [2], can be formulated as follows for an object moving on the OX axis only.

(8) $$\left[\begin{array}{c} x\left(k+1\right)\\ v_{x}\left(k+1\right) \end{array}\right]=\left[\begin{array}{cc} 1 & T\\ 0 & 1 \end{array}\right]\;\left[\begin{array}{c} x\left(k\right)\\ v_{x}\left(k\right) \end{array}\right]+\left[\begin{array}{c} w_{x}\left(k\right)\\ w_{v_{x}}\left(k\right) \end{array}\right],$$

where $w_{x}$ and $w_{v_{x}}$ are discrete disturbances acting on the object’s position and velocity, respectively.

It is, thus, a linear model, conforming with the general dynamics model Equation (1), where the state vector x, the transition matrix Φ, and the covariance matrix of process disturbances Q are as follows [2].

(9) $$x=\left[\begin{array}{c} x\\ v_{x} \end{array}\right],\;\Phi =\left[\begin{array}{cc} 1 & T\\ 0 & 1 \end{array}\right],\;Q=\left[\begin{array}{cc} \frac{S_{v_{x}}T^{3}}{3} & \frac{S_{v_{x}}T^{2}}{2}\\ \frac{S_{v_{x}}T^{2}}{2} & S_{v_{x}}T \end{array}\right],$$

where $S_{v_{x}}$ represents the power spectral density of continuous disturbances of the object’s motion.

The measurement model in the considered system is, on the other hand, non-linear, and can be written as follows, which conforms with the general measurement model of Equation (2).

(10) $$R\left(k\right)=h\left(x\left(k\right),v_{x}\left(k\right)\right)+\varepsilon_{R}\left(k\right)=\sqrt{{\left(x\left(k\right)-X_{0}\right)}^{2}+Y_{0}^{2}}+\varepsilon_{R}\left(k\right),$$

where $\varepsilon_{R}$ represents radar range measurement errors.

The measurement matrix H, calculated according to Equation (4) is as follows, where the *k* index was omitted for simplicity.

(11) $$H=\left[\begin{array}{cc} H_{11} & H_{12} \end{array}\right]=\left[\begin{array}{cc} \frac{\partial h\left(x,v_{x}\right)}{\partial x} & \frac{\partial h\left(x,v_{x}\right)}{\partial v_{x}} \end{array}\right]=\left[\begin{array}{cc} \frac{x-X_{0}}{\sqrt{{\left(x-X_{0}\right)}^{2}+Y_{0}^{2}}} & 0 \end{array}\right].$$

Since the measurement vector contains only one variable *R*, the covariance matrix of measurement errors is 1×1 and represents the variance of radar-object range measurements.

(12) $$R=\sigma_{R}^{2}.$$

The model presented above is enough to implement a standard algorithm of an extended Kalman filter. For the sake of its adaptive version, only a single scalar coefficient $M_{11}$ must be derived because only the $H_{11}$ element of the H matrix is non-zero. Moreover, it depends only on the first element *x* of the state vector x, which simplifies further calculations.

(13) $$\frac{\partial H_{11}}{\partial x}=\frac{\partial }{\partial x}\left(\frac{x-X_{0}}{\sqrt{{\left(x-X_{0}\right)}^{2}+Y_{0}^{2}}}\right)=\;\frac{Y_{0}^{2}}{{\left[{\left(x-X_{0}\right)}^{2}+Y_{0}^{2}\right]}^{3/2}},$$

(14) $$M_{11}\left(l\right)={\frac{\partial H_{11}}{\partial x}|}_{\hat{x}\left(l|l-1\right)}/H_{11}\left(l\right)=\frac{Y_{0}^{2}}{\left[\hat{x}\left(l|l-1\right)-X_{0}\right]\left[{\left(\hat{x}\left(l|l-1\right)-X_{0}\right)}^{2}+Y_{0}^{2}\right]}.$$

During the adaptive filter’s operation, at every time step k, the following condition is checked.

(15) $$\left|M_{11}\left(l\right)\left[\hat{x}\left(k+1|k\right)-\hat{x}\left(l|l-1\right)\right]\right|>\rho ,$$

Only if it is met, an updated value of $H_{11}$ is calculated.

When comparing numbers of operations and times of calculations in the standard EKF and in its modified version, their differences can be focused on. In the standard EKF, a calculation of $H_{11}$ is performed in every step k. Such a single calculation involves three additions, two multiplications, one division, and one calculation of the *sqrt*() function. In the proposed adaptive EKF, there is only one comparison, one addition, and one multiplication in every step k as well as six additions, six multiplications, two divisions, and one calculation of the *sqrt*() function in every step l. Therefore, the times of calculations of unique fragments of the measurement matrix updates for both filters can be expressed as follows.

(16) $$t_{upEKF1}=\sum_{k}\left(t_{add}\cdot n_{add1}+t_{mul}\cdot n_{mul1}+t_{div}\cdot n_{div1}+t_{sqrt}\cdot n_{sqrt1}\right)$$

(17) $$t_{upEKF2}=\sum_{k}\left(t_{comp}\cdot n_{comp2}+\;t_{add}\cdot n_{add2}+t_{mul}\cdot n_{mul2}\right)+\;+\sum_{l}\left(t_{add}\cdot n_{add_{l}}+t_{mul}\cdot n_{mul_{l}}+t_{div}\cdot n_{div_{l}}+t_{sqrt}\cdot n_{sqrt_{l}}\right)$$

where: $t_{upEKF1},t_{upEKF2}$ – total times of calculations of unique fragments of the measurement matrix updates in the standard EKF and in the modified EKF, respectively, $t_{comp},t_{add},t_{mul},t_{div},t_{sqrt}$ – times of single operations of comparison, addition, multiplication, division, and square root calculations, $n_{add1},n_{mul1},n_{div1},n_{sqrt1}$ – number of operations of addition, multiplication, division, and square-root in the standard EKF, $n_{comp2},n_{add2},n_{mul2}$ – number of operations of comparison, addition, and multiplication realized in all steps k in the modified EKF, $n_{add_{l}},n_{mul_{l}},n_{div_{l}},n_{sqrt_{l}}$ – number of operations of addition, multiplication, division, and square-root realized in the modified EKF in steps l only.

### 4.2. Angle-Only Tracking in 2D Radar

To demonstrate a possibility of using the proposed algorithm for a system with a different type of non-linearity, we reconsider the simple radar system presented in Figure 3. However, we assume that now only angles ϕ are measured. The previous assumption with respect to the radar location and the motion of the object remain unchanged.

The measurement model in this version of the system is as follows.

(18) $$\phi \left(k\right)=h\left(x\left(k\right),v_{x}\left(k\right)\right)+\varepsilon_{\phi }\left(k\right)=\mathrm{atan}2\left(-Y_{0},x\left(k\right)-X_{0}\right)+\varepsilon_{\phi }\left(k\right),$$

where $\;\varepsilon_{\phi }$ represents radar angle measurement errors.

The measurement matrix H, calculated according to Equation (4), is as follows.

(19) $$H=\left[\begin{array}{cc} H_{11} & H_{12} \end{array}\right]=\left[\begin{array}{cc} \frac{\partial h\left(x,v_{x}\right)}{\partial x} & \frac{\partial h\left(x,v_{x}\right)}{\partial v_{x}} \end{array}\right]=\left[\begin{array}{cc} \frac{Y_{0}}{{\left(x-X_{0}\right)}^{2}+Y_{0}^{2}} & 0 \end{array}\right],$$

The covariance matrix of measurement errors is 1×1 and contains the variance of angle measurements.

(20) $$R=\sigma_{\phi }^{2}.$$

For the sake of the adaptive filter, similarly to the range-only tracking problem, only a single scalar coefficient $M_{11}$ must be derived because only the $H_{11}$ element of the H matrix is non-zero.

(21) $$\frac{\partial H_{11}}{\partial x}=\frac{\partial }{\partial x}\left(\frac{Y_{0}}{{\left(x-X_{0}\right)}^{2}+Y_{0}^{2}}\right)=\;\frac{-2\left(x-X_{0}\right)Y_{0}}{{\left[{\left(x-X_{0}\right)}^{2}+Y_{0}^{2}\right]}^{2}}$$

(22) $$M_{11}\left(l\right)={\frac{\partial H_{11}}{\partial x}|}_{\hat{x}\left(l|l-1\right)}/H_{11}\left(l\right)=\frac{-2\left[\hat{x}\left(l|l-1\right)-X_{0}\right]}{{\left[\hat{x}\left(l|l-1\right)-X_{0}\right]}^{2}+Y_{0}^{2}}$$

The condition for updating the value of $H_{11}$ is the same as the previous one and it is given by Equation (15).

In the standard EKF for the considered system, a calculation of $H_{11}$ is performed in every step k and involves two additions, two multiplications, and one division. In the proposed adaptive EKF, there is only one comparison, one addition, and one multiplication in every step k, but, additionally, three additions, three multiplications, and one division in every step l. Therefore, the times of calculations of unique fragments of the measurement matrix updates for both filters can be expressed as follows.

(23) $$t_{upEKF1}=\sum_{k}\left(t_{add}\cdot n_{add1}+t_{mul}\cdot n_{mul1}+t_{div}\cdot n_{div1}\right)$$

(24) $$t_{upEKF2}=\sum_{k}\left(t_{comp}\cdot n_{comp2}+\;t_{add}\cdot n_{add2}+t_{mul}\cdot n_{mul2}\right)+\;+\sum_{l}\left(t_{add}\cdot n_{add_{l}}+t_{mul}\cdot n_{mul_{l}}+t_{div}\cdot n_{div_{l}}\right)$$

where: $t_{upEKF1},t_{upEKF2}$ – total times of calculations of unique fragments of the measurement matrix updates in the standard EKF and in the modified EKF, respectively, $t_{comp},t_{add},t_{mul},t_{div}$ – times of single operations of comparison, addition, multiplication, and division, $n_{add1},n_{mul1},n_{div1}$ – number of operations of addition, multiplication, and division in the standard EKF, $n_{comp2},n_{add2},n_{mul2}$ – number of operations of comparison, addition, and multiplication realized in all steps k in the modified EKF, and $n_{add_{l}},n_{mul_{l}},n_{div_{l}}$ – number of operations of addition, multiplication, and division realized in the modified EKF in steps l only.

### 4.3. Tracking in 2D Radar with Range and Angle Measurements

**Figure 4:**
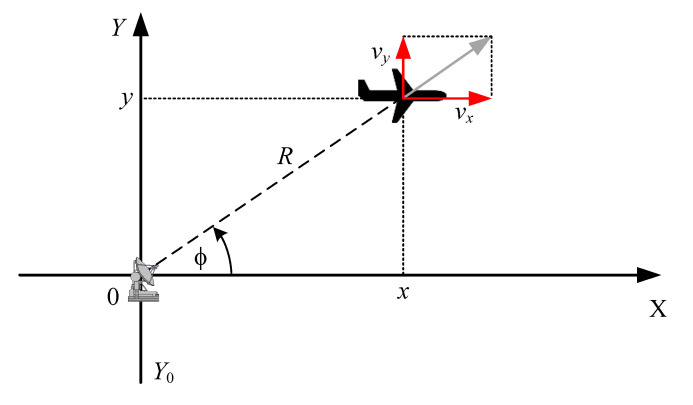
An example of a 2D radar system with range and angle measurements.

To provide a more general and practical example of the filter’s use, in this subsection, a 2D radar system processing both range and angle measurements is analyzed. The considered system is shown in Figure 4. It is assumed that the radar is located at the origin of the OXY frame of reference. The observed object can move in two directions, and the radar provides ranges *R* and angles ϕ.

A two-dimensional PV motion model for this system can be formulated as follows

(25) $$\left[\begin{array}{c} x\left(k+1\right)\\ \begin{array}{c} v_{x}\left(k+1\right)\\ y\left(k+1\right)\\ v_{y}\left(k+1\right) \end{array} \end{array}\right]=\left[\begin{array}{ccc} \begin{array}{c} 1\\ \begin{array}{c} 0\\ 0\\ 0 \end{array} \end{array} & \begin{array}{c} T\\ \begin{array}{c} 1\\ 0\\ 0 \end{array} \end{array} & \begin{array}{cc} \begin{array}{c} 0\\ \begin{array}{c} 0\\ 1\\ 0 \end{array} \end{array} & \begin{array}{c} 0\\ \begin{array}{c} 0\\ T\\ 1 \end{array} \end{array} \end{array} \end{array}\right]\cdot \left[\begin{array}{c} x\left(k\right)\\ \begin{array}{c} v_{x}\left(k\right)\\ y\left(k\right)\\ v_{y}\left(k\right) \end{array} \end{array}\right]+\left[\begin{array}{c} w_{x}\left(k\right)\\ \begin{array}{c} w_{v_{x}}\left(k\right)\\ w_{y}\left(k\right)\\ w_{v_{y}}\left(k\right) \end{array} \end{array}\right],$$

where $w_{x}$, $w_{v_{x}}$, $w_{y},$ and $w_{v_{y}}$ are discrete disturbances acting on the object’s position and velocity, respectively.

The state vector x, the transition matrix Φ, and the covariance matrix of process disturbances Q are as follows.

(26) $$x=\left[\begin{array}{c} x\\ \begin{array}{c} v_{x}\\ y\\ v_{y} \end{array} \end{array}\right],\;\Phi =\left[\begin{array}{ccc} \begin{array}{c} 1\\ \begin{array}{c} 0\\ 0\\ 0 \end{array} \end{array} & \begin{array}{c} T\\ \begin{array}{c} 1\\ 0\\ 0 \end{array} \end{array} & \begin{array}{cc} \begin{array}{c} 0\\ \begin{array}{c} 0\\ 1\\ 0 \end{array} \end{array} & \begin{array}{c} 0\\ \begin{array}{c} 0\\ T\\ 1 \end{array} \end{array} \end{array} \end{array}\right]Q=\left[\begin{array}{c} \frac{S_{v_{x}}T^{3}}{3}\\ \begin{array}{c} \frac{S_{v_{x}}T^{2}}{2}\\ 0\\ 0 \end{array} \end{array}\begin{array}{c} \frac{S_{v_{x}}T^{2}}{2}\\ \begin{array}{c} S_{v_{x}}T\\ 0\\ 0 \end{array} \end{array}\begin{array}{c} 0\\ \begin{array}{c} 0\\ \frac{S_{v_{y}}T^{3}}{3}\\ \frac{S_{v_{y}}T^{2}}{2} \end{array} \end{array}\begin{array}{c} 0\\ \begin{array}{c} 0\\ \frac{S_{v_{y}}T^{2}}{2}\\ S_{v_{y}}T \end{array} \end{array}\right],$$

where $S_{v_{x}}$ and $S_{v_{y}}$ represent the power spectral densities of disturbances of the object’s motion.

The measurement model in the considered system is as follows.

(27) $$\left[\begin{array}{c} R\left(k\right)\\ \phi \left(k\right) \end{array}\right]=\left[\begin{array}{c} h_{1}\left(x\left(k\right),v_{x}\left(k\right),y\left(k\right),v_{x}\left(k\right)\right)\\ h_{2}\left(x\left(k\right),v_{x}\left(k\right),y\left(k\right),v_{x}\left(k\right)\right) \end{array}\right]+\left[\begin{array}{c} v_{R}\left(k\right)\\ v_{\phi }\left(k\right) \end{array}\right]=\left[\begin{array}{c} \sqrt{x{\left(k\right)}^{2}+y{\left(k\right)}^{2}}\\ \mathrm{atan}2\left(y\left(k\right),x\left(k\right)\right) \end{array}\right]+\left[\begin{array}{c} v_{R}\left(k\right)\\ v_{\phi }\left(k\right) \end{array}\right],$$

where $v_{R}$ and $v_{\phi }$ represent radar range and angle measurement errors.

The measurement matrix H, calculated according to Equation (4), is as follows, where the *k* index was omitted for simplicity.

(28) $$H=\left[\begin{array}{cc} \begin{array}{c} H_{11}\\ H_{21} \end{array} & \begin{array}{ccc} \begin{array}{c} H_{12}\\ H_{22} \end{array} & \begin{array}{c} H_{13}\\ H_{23} \end{array} & \begin{array}{c} H_{14}\\ H_{24} \end{array} \end{array} \end{array}\right]=\left[\begin{array}{c} \frac{\partial h_{1}\left(x,v_{x},y,v_{y}\right)}{\partial x}\\ \frac{\partial h_{2}\left(x,v_{x},y,v_{y}\right)}{\partial x} \end{array}\begin{array}{c} \frac{\partial h_{1}\left(x,v_{x},y,v_{y}\right)}{\partial v_{x}}\\ \frac{\partial h_{2}\left(x,v_{x},y,v_{y}\right)}{\partial v_{x}} \end{array}\begin{array}{c} \frac{\partial h_{1}\left(x,v_{x},y,v_{y}\right)}{\partial y}\\ \frac{\partial h_{2}\left(x,v_{x},y,v_{y}\right)}{\partial y} \end{array}\begin{array}{c} \frac{\partial h_{1}\left(x,v_{x},y,v_{y}\right)}{\partial v_{y}}\\ \frac{\partial h_{2}\left(x,v_{x},y,v_{y}\right)}{\partial v_{y}} \end{array}\right]\left[\begin{array}{cc} \begin{array}{c} \frac{x}{\sqrt{x^{2}+y^{2}}}\\ \frac{-y}{x^{2}+y^{2}} \end{array} & \begin{array}{ccc} \begin{array}{c} 0\\ 0 \end{array} & \begin{array}{c} \frac{y}{\sqrt{x^{2}+y^{2}}}\\ \frac{x}{x^{2}+y^{2}} \end{array} & \begin{array}{c} 0\\ 0 \end{array} \end{array} \end{array}\right].$$

The covariance matrix of measurement errors contains the variances of range and angle measurements.

(29) $$R=\left[\begin{array}{cc} \sigma_{R}^{2} & 0\\ 0 & \sigma_{\phi }^{2} \end{array}\right].$$

The coefficients $M_{ij}\left(l\right)$ must be calculated according to Equation (7) for the non-zero H matrix elements, and, since these elements are functions of two variables x and y, the mentioned coefficients are vectors rather than scalars. The way of calculation of the $M_{ij}\left(l\right)$ coefficients is explained below.

(30) $$\frac{\partial H_{11}}{\partial x}=\frac{\partial }{\partial x}\left(\frac{x}{\sqrt{x^{2}+y^{2}}}\right)=\;\frac{y^{2}}{{\left[x^{2}+y^{2}\right]}^{3/2}},$$

(31) $$\frac{\partial H_{11}}{\partial y}=\frac{\partial }{\partial y}\left(\frac{x}{\sqrt{x^{2}+y^{2}}}\right)=\;\frac{-xy}{{\left[x^{2}+y^{2}\right]}^{3/2}},$$

(32) $$\frac{\partial H_{13}}{\partial x}=\frac{\partial }{\partial x}\left(\frac{y}{\sqrt{x^{2}+y^{2}}}\right)=\;\frac{-xy}{{\left[x^{2}+y^{2}\right]}^{3/2}},$$

(33) $$\frac{\partial H_{13}}{\partial y}=\frac{\partial }{\partial y}\left(\frac{y}{\sqrt{x^{2}+y^{2}}}\right)=\;\frac{x^{2}}{{\left[x^{2}+y^{2}\right]}^{3/2}},$$

(34) $$\frac{\partial H_{21}}{\partial x}=\frac{\partial }{\partial x}\left(\frac{-y}{x^{2}+y^{2}}\right)=\;\frac{2xy}{{\left(x^{2}+y^{2}\right)}^{2}},$$

(35) $$\frac{\partial H_{21}}{\partial y}=\frac{\partial }{\partial y}\left(\frac{-y}{x^{2}+y^{2}}\right)=\;\frac{y^{2}-x^{2}}{{\left(x^{2}+y^{2}\right)}^{2}},$$

(36) $$\frac{\partial H_{23}}{\partial x}=\frac{\partial }{\partial x}\left(\frac{x}{x^{2}+y^{2}}\right)=\;\frac{y^{2}-x^{2}}{{\left(x^{2}+y^{2}\right)}^{2}},$$

(37) $$\frac{\partial H_{23}}{\partial y}=\frac{\partial }{\partial y}\left(\frac{x}{x^{2}+y^{2}}\right)=\;\frac{-2xy}{{\left(x^{2}+y^{2}\right)}^{2}},$$

(38) $$M_{11}\left(l\right)=\left[\begin{array}{ccc} \frac{\hat{y}{\left(l|l-1\right)}^{2}}{\hat{x}\left(l|l-1\right)\left[\hat{x}{\left(l|l-1\right)}^{2}+\hat{y}{\left(l|l-1\right)}^{2}\right]} & 0 & \begin{array}{cc} \frac{-\hat{y}\left(l|l-1\right)}{\hat{x}{\left(l|l-1\right)}^{2}+\hat{y}{\left(l|l-1\right)}^{2}} & 0 \end{array} \end{array}\right],$$

(39) $$M_{13}\left(l\right)=\left[\begin{array}{ccc} \frac{-\hat{x}\left(l|l-1\right)}{\hat{x}{\left(l|l-1\right)}^{2}+\hat{y}{\left(l|l-1\right)}^{2}} & 0 & \begin{array}{cc} \frac{\hat{x}{\left(l|l-1\right)}^{2}}{\hat{y}\left(l|l-1\right)\left[\hat{x}{\left(l|l-1\right)}^{2}+\hat{y}{\left(l|l-1\right)}^{2}\right]} & 0 \end{array} \end{array}\right],$$

(40) $$M_{21}\left(l\right)=\left[\begin{array}{ccc} \frac{-2\hat{x}\left(l|l-1\right)}{\hat{x}{\left(l|l-1\right)}^{2}+\hat{y}{\left(l|l-1\right)}^{2}} & 0 & \begin{array}{cc} \frac{\hat{x}{\left(l|l-1\right)}^{2}-\hat{y}{\left(l|l-1\right)}^{2}}{\hat{y}\left(l|l-1\right)\left[\hat{x}{\left(l|l-1\right)}^{2}+\hat{y}{\left(l|l-1\right)}^{2}\right]} & 0 \end{array} \end{array}\right],$$

(41) $$M_{23}\left(l\right)=\left[\begin{array}{ccc} \frac{\hat{x}{\left(l|l-1\right)}^{2}-\hat{y}{\left(l|l-1\right)}^{2}}{\hat{y}\left(l|l-1\right)\left[\hat{x}{\left(l|l-1\right)}^{2}+\hat{y}{\left(l|l-1\right)}^{2}\right]} & 0 & \begin{array}{cc} \frac{2\hat{x}\left(l|l-1\right)}{\hat{x}{\left(l|l-1\right)}^{2}+\hat{y}{\left(l|l-1\right)}^{2}} & 0 \end{array} \end{array}\right].$$

The following four conditions are checked at every time step k during the filter’s operation.

(42) $$\left|M_{11}\left(l\right)\left[\hat{x}\left(k+1|k\right)-\hat{x}\left(l|l-1\right)\right]\right|>\rho_{11},$$

(43) $$\left|M_{13}\left(l\right)\left[\hat{x}\left(k+1|k\right)-\hat{x}\left(l|l-1\right)\right]\right|>\rho_{13},$$

(44) $$\left|M_{21}\left(l\right)\left[\hat{x}\left(k+1|k\right)-\hat{x}\left(l|l-1\right)\right]\right|>\rho_{21},$$

(45) $$\left|M_{23}\left(l\right)\left[\hat{x}\left(k+1|k\right)-\hat{x}\left(l|l-1\right)\right]\right|>\rho_{23},$$

From Equations (38)–(41), it is visible that the above conditions can be reduced to simpler scalar inequalities. The respective element of the H matrix, i.e., $H_{11},\;H_{13},H_{21},$ or $H_{23}$ is updated only if one of the above conditions is met, i.e., one of the thresholds $\rho_{11},\rho_{13},\rho_{21},$ or $\rho_{23}$ is exceeded.

## 5. Results of Algorithm Testing

The proposed modified EKF algorithm was implemented and tested for all examples of systems described in Section 4 and the obtained results are shown in subsequent subsections.

### 5.1. Range-Only Tracking Radar Simulations

The results presented in this subsection refer to the EKF described in Section 4.1, which was designed for the 2D radar with range-only measurements available. The following conditions were assumed for these simulations.

- Simulation time of 100 seconds,
- Period of measurements and filter date T=1 s,
- Radar coordinates $X_{0}=5000\;m$, $Y_{0}=-1000\;m$,
- Object moving in the positive direction of the OX axis, with the initial position, velocity, and acceleration equal: x(0)=1000 m, $v_{x}\left(0\right)=100\;\frac{m}{s}$, $a_{x}\left(0\right)=0\;\frac{m}{s^{2}}$, respectively,
- Power spectral density of the white noise of the motion disturbances $S_{v_{x}}=0.1\;\frac{m^{2}}{s^{3}}$,
- Standard deviation of range measurements $\sigma_{R}=10\;m$.

In the conducted tests of the algorithm, the threshold ρ was changed in the range from 0 to 0.1 with a step 0.01, and its influence on the number of the measurement matrix H updates and on the root-mean-squared (RMS) estimation error of the x coordinate of the object’s position. Since the results for various runs of simulations are variable due to various realizations of process disturbances and measurement errors, their values were averaged for 100 thousand simulations for each considered threshold value. The obtained values are gathered in Table 1. Due to the mentioned averaging, the number of H matrix updates in this table is not an integer number.

**Figure 5:**
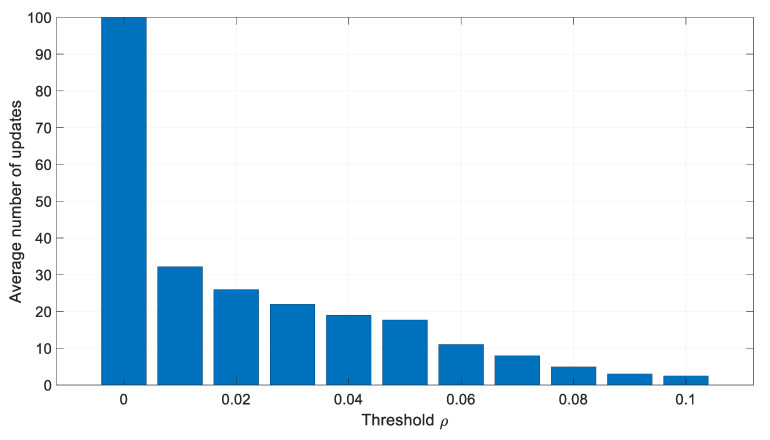
Influence of the threshold ρ on the number of measurement matrix updates.

**Figure 6:**
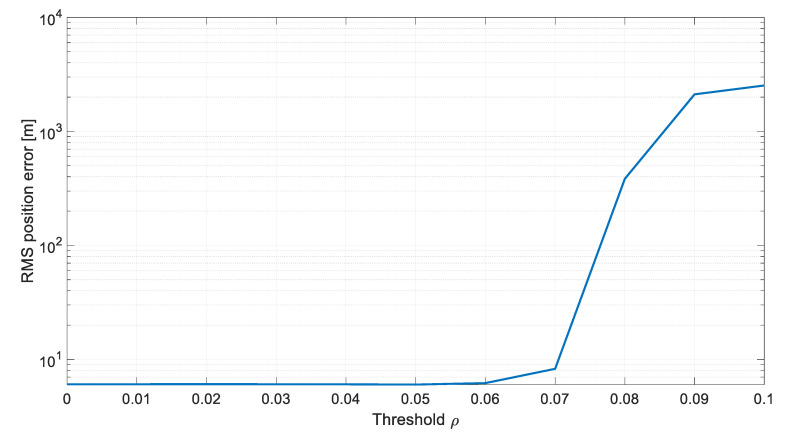
Influence of the threshold ρ on the RMS errors of position estimation.

The results from Table 1 are also graphically presented in Figure 5 and Figure 6.

In order to verify what is the influence of the intensity of the measurement errors on the optimal threshold value, the following experiment was conducted. For two different standard deviations of range errors, i.e., $\sigma_{R}\;=1\;m$ and $\sigma_{R}\;=100\;m$ the threshold ρ was changed in the range from 0 to 0.8 with a step 0.01, and its influence on the number of the measurement matrix H updates and on the root-mean-squared estimation error of the x coordinate of the object’s position were registered. The respective results are gathered in Table 2 and Table 3. Even for significantly (an order of magnitude) stronger or weaker measurement noises, the same threshold ρ=0.06 gives the best results. Thus, there is no need for adaptive threshold changes in the modified EKF even if one expects that the noise level may be variable.

Computation loads and times for arithmetic operations are largely dependent on the processor used and the implementation of functions in the applied library. However, it is generally assumed that comparisons and additions are the least computationally expensive, and more demanding, in increasing order, are multiplications, divisions, and calculations of functions, such as the square-root or trigonometric functions [17]. Assume for illustrative purposes the times of elementary operations given in Reference [17] for a specific computer, namely the IBM 360 Model 67-1. They are 2.7 μs for addition, 4.1 μs for multiplication, 6.6 μs for division, and 60 μs for the square root, respectively. Assume that the time for comparison equals that of adding two variables. The times of the calculations of unique fragments of the measurement matrix updates in the standard EKF and in the modified EKF, calculated according to Equations (16) and (17) for the considered example, are gathered in Table 4.

The results presented in Table 4 show significant savings in terms of processing time for the adopted assumptions, even for small thresholds ρ where the H matrix updates are still frequently performed.

**Figure 7:**
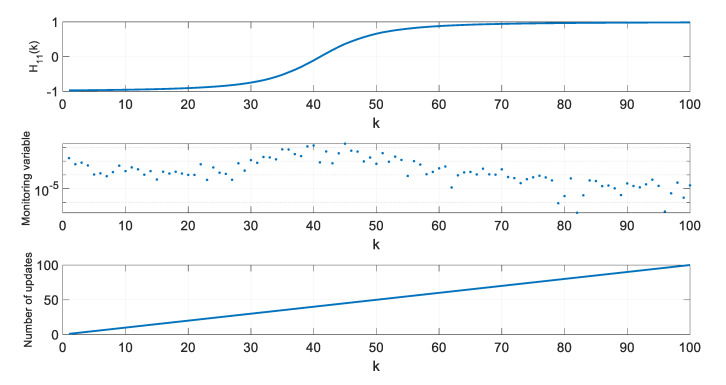
Values of $H_{11}$, $M_{11}\left(l\right)\left[\hat{x}\left(k+1|k\right)-\hat{x}\left(l|l-1\right)\right],$ and number of H matrix updates for ρ=0.

**Figure 8:**
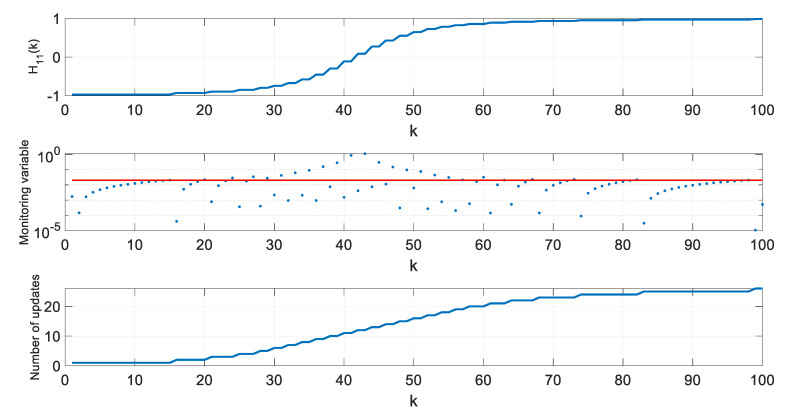
Values of $H_{11}$, $M_{11}\left(l\right)\left[\hat{x}\left(k+1|k\right)-\hat{x}\left(l|l-1\right)\right],$ and number of H matrix updates for ρ=0.02.

**Figure 9:**
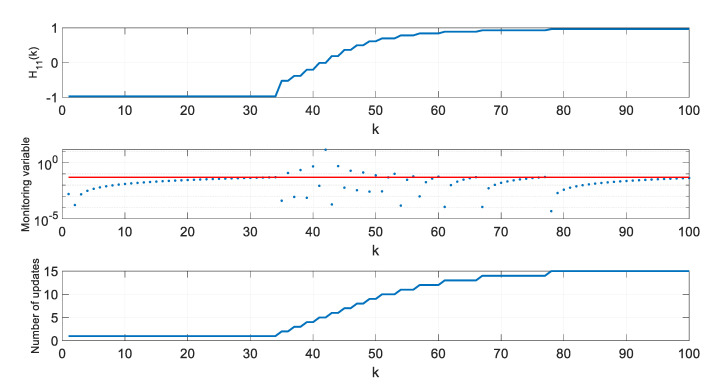
Values of $H_{11}$, $M_{11}\left(l\right)\left[\hat{x}\left(k+1|k\right)-\hat{x}\left(l|l-1\right)\right]$, and number of H matrix updates for ρ=0.05.

**Figure 10:**
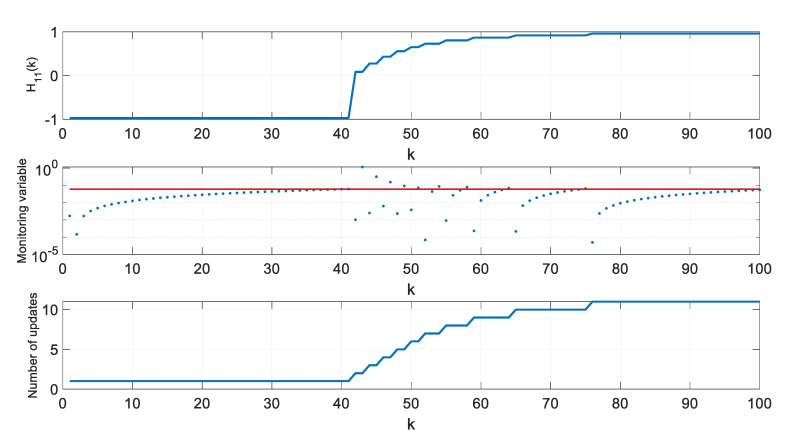
Values of $H_{11}$, $M_{11}\left(l\right)\left[\hat{x}\left(k+1|k\right)-\hat{x}\left(l|l-1\right)\right]$, and number of H matrix updates for ρ=0.06.

In a further step of algorithm testing, for four selected thresholds ρ, values of the $H_{11}$ element of the H matrix, values of the monitoring variable $\left|M_{11}\left(l\right)\left[\hat{x}\left(k+1|k\right)-\hat{x}\left(l|l-1\right)\right]\right|$, and the number of **H** matrix updates since the beginning of a single simulation were calculated and are presented in Figure 7, Figure 8, Figure 9 and Figure 10.

In the presented figures, one can see that the number of H matrix updates decreases with the increasing threshold ρ value.

The case ρ=0 is equivalent to a standard EKF with the measurement matrix update taking place in every step k, whereas the case from Figure 10, obtained for ρ=0.06, represents a situation when the position estimation accuracy is still almost the same as in a standard EKF, but the filter’s computational demands are significantly reduced.

With increasing threshold value, the number of H matrix updates decreases, but their uneven distribution in time is worth noting. The largest density of actualizations takes place in a portion of the object’s trajectory, where the cosine of the ϕ angle, under which the object is seen by the radar, changes most rapidly. Its quickest changes are in the central part of the assumed flight trajectory, and they result from the fact that

(46) $$H_{11}=\frac{x-X_{0}}{\sqrt{{\left(x-X_{0}\right)}^{2}+Y_{0}^{2}}}=\cos\left(\phi \right)$$

changes most rapidly near $\phi =90^{o}$.

**Figure 11:**
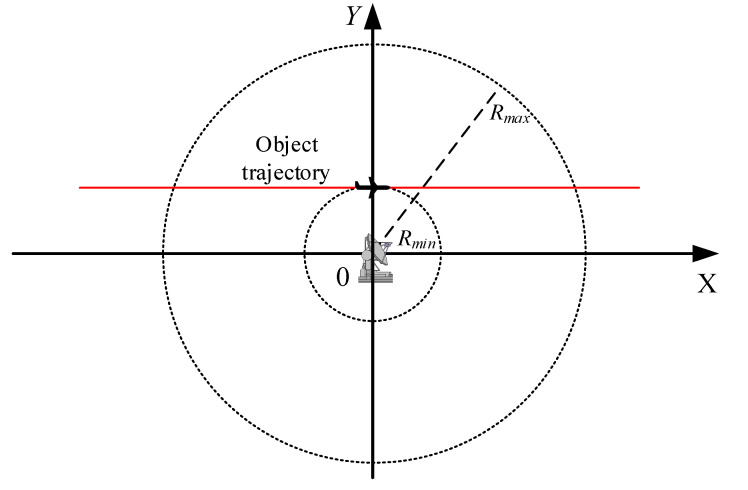
Trajectory used for establishing the threshold ρ by a simulation method.

This notion leads to establishing a practical method for choosing the threshold. The threshold ρ should be chosen for a specific radioelectronic system, based on simulations or on analytical considerations. One can considered “the worst case” scenario where the H matrix elements change the quickest. In case of a radar system, with known minimal detection range $R_{min},$ the quickest H matrix updates are required for an object passing the radar in such a small distance, as presented in Figure 11, and the threshold ρ can be established for this specific case only, looking for its maximal value, but still ensuring acceptable estimation accuracy.

For better visualization of the filter’s behavior, the presented example was purposefully chosen in such a way that the H matrix changes significantly in the short time of simulation. In most radar and radio-navigation systems, these changes would be much slower. For example, in the Kalman filter of a Global Positioning System (GPS) receiver, the H matrix contains direction cosines from the user to the visible GPS satellites [2]. Since the angles under which the satellites are seen from a user’s location remain almost unchanged in short periods of time due to the very large distances between the user and the satellites, the H matrix elements would be almost constant for relatively long periods. In such a filter, the H matrix updates would be performed very rarely in comparison to the radar system example that was considered in this paper. Therefore, in real radioelectronic systems, the proposed EKF modification could lead to an even more significant reduction of its computational demands.

### 5.2. Angle-Only Tracking Radar Simulations

The results presented in this subsection of the paper refer to the EKF described in Section 4.2 designed for the 2D radar with angle-only measurements available. The conditions adopted for these simulations were the same as for the range-only tracking radar, with a single difference, consisting of assuming a standard deviation of angle measurements $\sigma_{\phi }=0.1{}^{\circ}$ instead of a standard deviation of range measurements.

In the first test of the algorithm, the threshold ρ was changed in the range from 0 to 0.1 with a step 0.02 and from 0.1 to 0.2 with a step 0.05. Its influence on the number of the measurement matrix H updates and on the root-mean-squared estimation error of the x coordinate of the object’s position was analyzed and gathered in Table 5. The decreasing number of matrix updates does not visibly affect the estimation error.

The times of the calculations of unique fragments of the measurement matrix updates in the standard EKF and in the modified EKF, calculated according to Equations (23) and (24) for the considered example, are gathered in Table 6.

Similar to the range-only tracking, in this case, computational savings can be obtained. However, they are not as large as previously seen. This is due to a fact that, in the range-only tracking problem, the $H_{11}$ element of the H matrix contained a time-consuming square root operation and its omitting in some steps immediately resulted in a large decrease of the processing time. In the angle-only tracking, the $H_{11}$ element is a simpler function that can be decomposed into a few additions, multiplications, and divisions. Thus, it is not computationally expensive. The savings are still possible only due to limiting the number of the mentioned elementary operations.

Based on the already presented examples, one can conclude that the proposed modified filter would be most effective in problems where the H matrix elements are computationally expensive so that the obtained savings due to less frequent H matrix updates are much larger than the additional computational burden due to calculations of monitoring variables and their comparisons with a threshold. As the EKFs used in GPS receivers process pseudoranges closely related to ranges, and typical radars process both ranges and angles, the H matrix elements in these filters contain time-consuming operations, like square roots. Therefore, the proposed filter may be useful in these important groups of applications.

**Figure 12:**
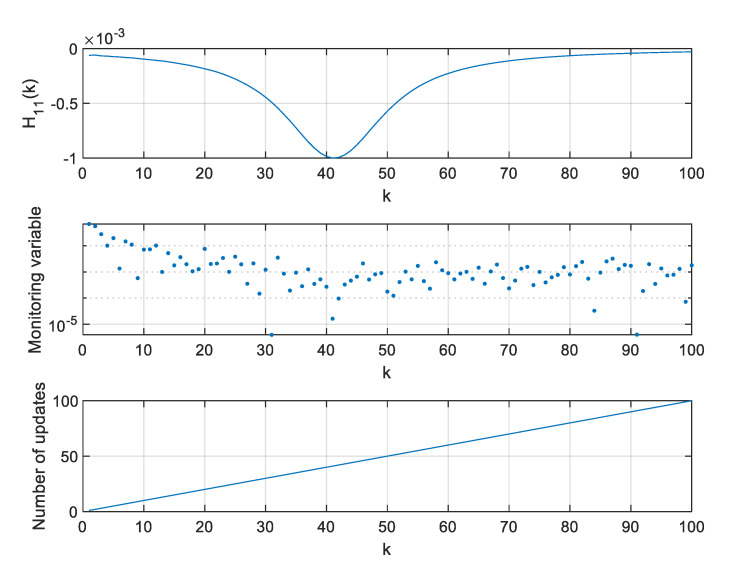
Values of $H_{11}$, $M_{11}\left(l\right)\left[\hat{x}\left(k+1|k\right)-\hat{x}\left(l|l-1\right)\right]$ and number of H matrix updates for ρ=0.

**Figure 13:**
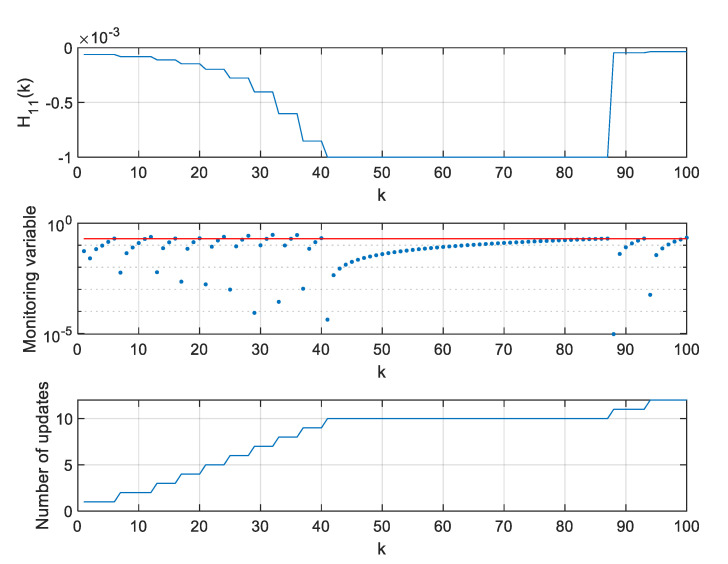
Values of $H_{11}$, $M_{11}\left(l\right)\left[\hat{x}\left(k+1|k\right)-\hat{x}\left(l|l-1\right)\right]$ and number of H matrix updates for ρ=0.2.

**Figure 14:**
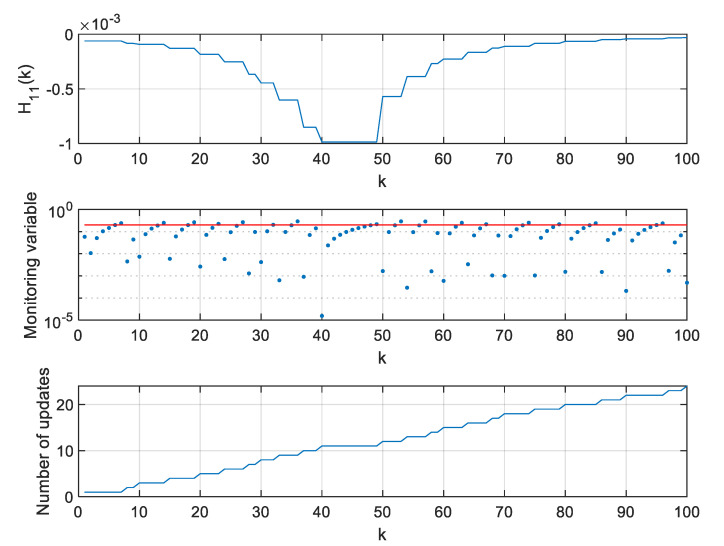
Values of $H_{11}$, $M_{11}\left(l\right)\left[\hat{x}\left(k+1|k\right)-\hat{x}\left(l|l-1\right)\right]$ and number of H matrix updates for ρ=0.2 in the Extended Kalman filter (EKF) with a forced H matrix update every 10 steps.

For two extreme thresholds ρ, values of the $H_{11}$ element of the H matrix, values of the monitoring variable $\left|M_{11}\left(l\right)\left[\hat{x}\left(k+1|k\right)-\hat{x}\left(l|l-1\right)\right]\right|$, and the number of **H** matrix updates since the beginning of a single simulation were calculated and are presented in Figure 12, Figure 13 and Figure 14.

As can be seen in Figure 12, the $H_{11}$ element of the H matrix reaches its minimum at step 41 and then very rapidly changes its direction. This results from a very small distance between the radar and the object in the considered case. It should be mentioned that this is not a typical situation in real systems where EKFs are used, as the EKF itself is dedicated for systems with relatively benign non-linearities [10]. In strongly non-linear systems, Unscented Kalman Filters or Particle Filters are typically applied [9,11].

However, the effects observed in the considered scenario give some insight into the algorithm’s behavior. If the value of $H_{11}$ derivative is calculated around step k=40, it will be close to zero and, consequently, the variable $M_{11}$ will also be close to zero. In such a case, the proposed algorithm may be too sluggish, and it will wait long with the next update of the H matrix. This effect can be observed in Figure 13 obtained for a large ρ=0.2.

If rapid changes of the H matrix elements occur in a system for which the proposed algorithm is designed, one can consider forcing the update of the H matrix not only based on the observation of the monitoring variables, but also a predefined number of steps, whichever happens first. The results obtained for the above described filter but with a forced H matrix update every 10 steps are shown in Figure 14. It is visible that such a filter can effectively work in high-response conditions and deal with its sluggish behavior when the H matrix elements reach their extreme values and their derivatives are close to zeroes.

### 5.3. 2D Radar with Range and Angle Measurement Simulations

The results presented in this subsection refer to the EKF described in Section 4.3, processing both range and angle measurements in a 2D radar. The following conditions were assumed for these simulations.

- Simulation time 100 s,
- Period of measurements and filter update T=1 s,
- Radar coordinates $X_{0}=0\;m$, $Y_{0}=0\;m$,
- Object moving from the initial position x(0)=−5000 m, y(0)=5000 m, with the initial velocity $v_{x}\left(0\right)=100\;\frac{m}{s}$, $v_{y}\left(0\right)=-10\;\frac{m}{s}$, and the initial acceleration $a_{x}\left(0\right)=a_{y}\left(0\right)=0\;\frac{m}{s^{2}}$,
- Power spectral density of the white noise of the motion disturbances $S_{v_{x}}=S_{v_{y}}=0.1\;\frac{m^{2}}{s^{3}}$,
- Standard deviation of range measurements $\sigma_{R}=100\;m$,
- Standard deviation of angle measurements $\sigma_{\phi }=1{}^{\circ}$.

To demonstrate the results obtained with a standard EKF and the proposed modified filter version, a trajectory of a moving object and pairs of range-angle measurements were generated. In the modified Kalman filter, a threshold was set to ρ=0.06. The object trajectory estimated by both filters is compared in Figure 13. It is visible that, despite the limited number of the H matrix updates, the results of estimation from the modified Kalman filter are similar to those from a standard EKF.

**Figure 15:**
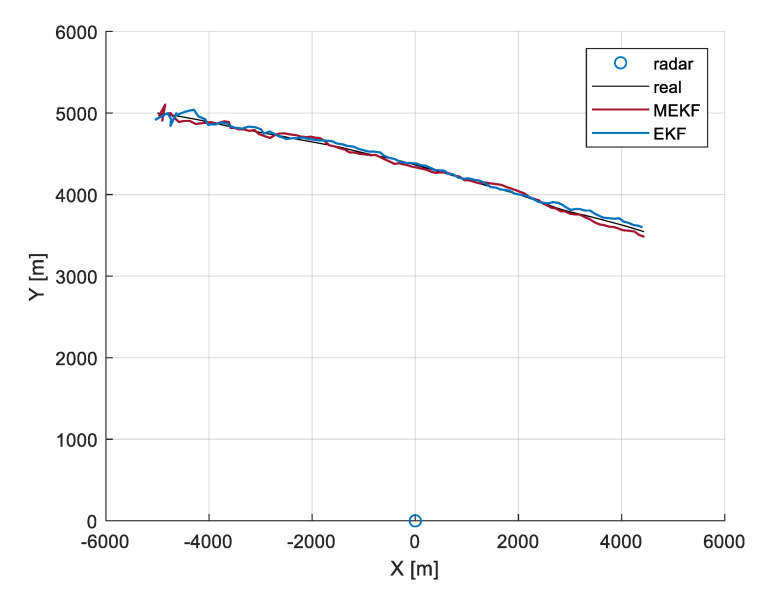
Comparison of trajectories estimated with a standard EKF and modified EKF (MEKF).

**Figure 16:**
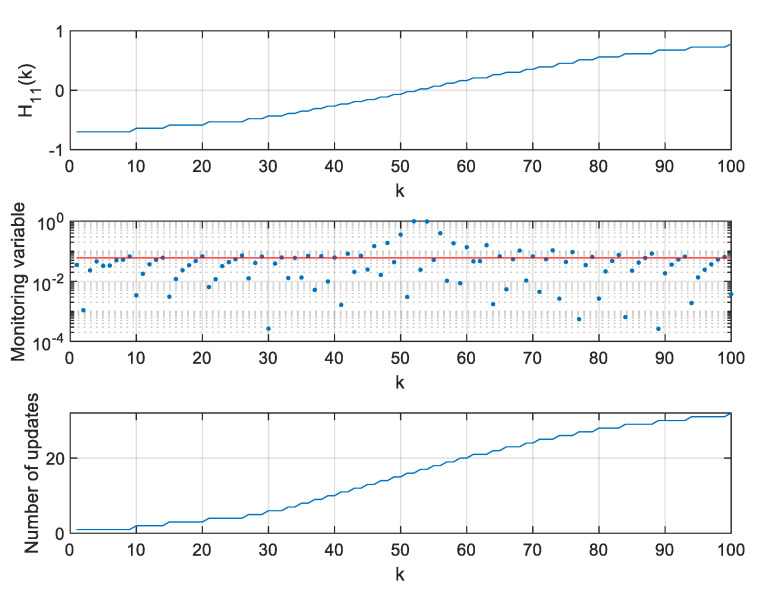
Values of $H_{11}$, monitoring variable, and number of $H_{11}$ element updates for ρ=0.06.

**Figure 17:**
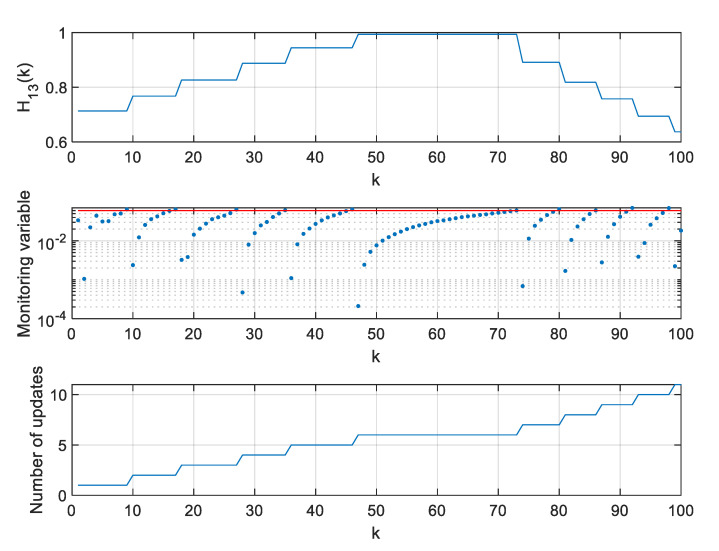
Values of $H_{13}$, monitoring variable, and number of $H_{13}$ element updates for ρ=0.06.

**Figure 18:**
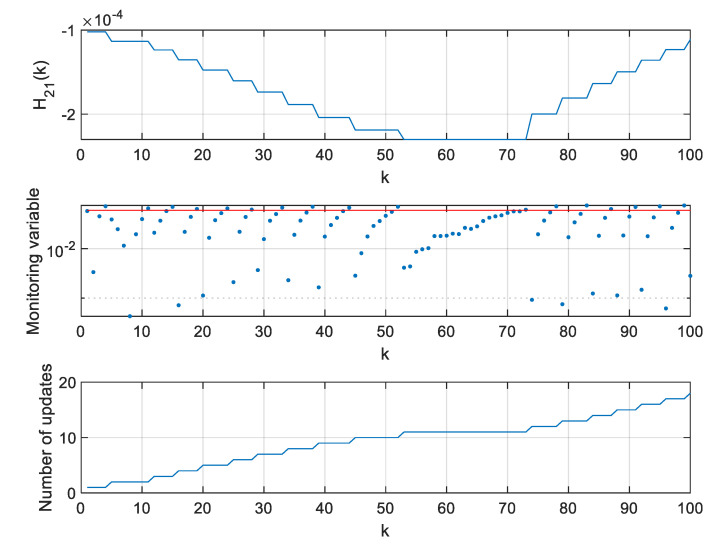
Values of $H_{21}$, monitoring variable, and number of $H_{21}$ element updates for ρ=0.06.

**Figure 19:**
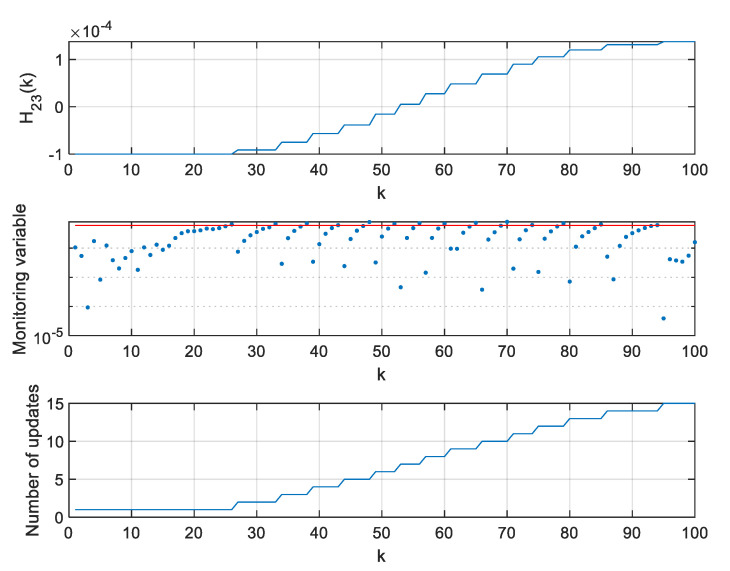
Values of $H_{23}$, monitoring variable, and number of $H_{23}$ element updates for ρ=0.06.

The values of $H_{11},H_{13},H_{21},$ and $H_{23}$ elements of the H matrix, values of respective monitoring variables, and the number of $H_{11},H_{13},H_{21},$ and $H_{23}$ elements’ updates since the beginning of the simulation are presented in Figure 15, Figure 16, Figure 17, Figure 18 and Figure 19.

## 6. Conclusions

The paper presented a classical Extended Kalman Filter and its modified version with a reduced computational burden due to the omission of unnecessary H matrix updates. The effects of the proposed modification were analyzed for a simple radar system model. Two simple cases of range-only and angle-only tracking were analyzed in detail and a more general and practical example of a 2D radar system processing both range and angle measurements was demonstrated.

In the case of range-only tracking, for the assumed threshold ρ=0.06, the accuracy of the object position estimation remained almost unaffected despite the number of the H matrix updates being reduced to only 11% of the number in a standard EKF. The presented simulation results show that initially increasing the threshold ρ value does not change the filter’s accuracy, but, at the same time, decreases its number of computations. When exceeding a specific threshold value, ρ=0.07 in the considered example, estimation errors increase rapidly to a very large and unacceptable level.

In the case of angle-only tracking, the computational savings can also be achieved. However, they are not as prominent as in the previous system, which results from a simpler and less computationally demanding function in the H matrix. The comparison of these two cases leads to a conclusion that the proposed adaptive filter would be most effective in problems where the H matrix elements are complicated and computationally demanding. Then, significant savings on the number of operations can be achieved with an even slight reduction of the number of the H matrix updates.

The proposed estimation method can be used in any field where the estimation of a state vector in a non-linear system is necessary, but the applications presented in this paper are related mainly to radiolocation and radio-navigation. It seems that, as the EKFs used in GNSS receivers process observables related to ranges, and typical radars process both ranges and angles, the H matrix elements in these filters contain enough time-consuming operations, that applying the proposed filter in them could be beneficial.

## Figures and Tables

**Table 1:** Influence of the threshold ρ on the number of the measurement matrix updates and the estimation accuracy.

Threshold	Average Number of Updates	RMS Position Error [m]
0	100	6.045
0.01	32.20	6.054
0.02	25.96	6.063
0.03	22.00	6.053
0.04	19.00	6.042
0.05	17.71	6.013
0.06	11.04	6.195
0.07	7.998	8.265
0.08	4.977	383.9
0.09	3.039	2115
0.1	2.467	2530

**Table 2:** Influence of the threshold ρ on the number of the measurement matrix updates and the estimation accuracy for a decreased intensity of the measurement errors $\sigma_{R}\;=1\;m$.

Threshold	Average Number of Updates	RMS Position Error [m]
0	100	1.877
0.01	31.00	1.887
0.02	26.00	1.886
0.03	22.00	1.887
0.04	19.00	1.885
0.05	16.00	1.895
**0.06**	**11.00**	**2.041**
0.07	8.00	3.214
0.08	5.00	10.32

**Table 3:** Influence of the threshold ρ on the number of the measurement matrix updates and the estimation accuracy for an increased intensity of the measurement errors $\sigma_{R}\;=100\;m.$

Threshold	Average Number of Updates	RMS Position Error [m]
0	100	57.89
0.01	38.97	57.89
0.02	30.44	57.88
0.03	25.49	57.92
0.04	21.52	57.81
0.05	17.12	57.39
0.06	11.72	58.83
0.07	6.963	1822
0.08	3.461	3151

**Table 4:** Influence of the threshold ρ on the times of calculations of unique fragments of the measurement matrix updates.

Threshold	Standard EKF $\left(t_{upEKF1}\;\left[\mathrm{ms}\right]\right)$	Modified EKF $\left(t_{upEKF2}\;\left[\mathrm{ms}\right]\right)$
0	8.29	12.3
0.01	8.29	4.62
0.02	8.29	3.91
0.03	8.29	3.46
0.04	8.29	3.11
0.05	8.29	2.97
0.06	8.29	2.21
0.07	8.29	1.86
0.08	8.29	1.52
0.09	8.29	1.30
0.1	8.29	1.23

**Table 5:** Influence of the threshold ρ on the number of the measurement matrix updates and the estimation accuracy.

Threshold	Average Number of Updates	RMS Position Error [m]
0	100	8.894
0.02	49.89	8.909
0.04	46.24	8.911
0.06	39.03	8.907
0.08	35.02	8.886
0.1	28.68	8.893
0.15	21.28	8.830
0.20	18.07	8.889

**Table 6:** Influence of the threshold ρ on the times of calculations of unique fragments of the measurement matrix updates.

Threshold	Standard EKF $\left(t_{upEKF1}\;\left[\mathrm{ms}\right]\right)$	Modified EKF $\left(t_{upEKF2}\;\left[\mathrm{ms}\right]\right)$
0	2.02	3.65
0.02	2.02	2.30
0.04	2.02	2.20
0.06	2.02	2.00
0.08	2.02	1.90
0.1	2.02	1.72
0.15	2.02	1.52
0.20	2.02	1.44
